# Subcutaneous Bacillus Calmette–Guérin Administration Induces Innate Training in Monocytes in Preweaned Holstein Calves

**DOI:** 10.4049/immunohorizons.2300047

**Published:** 2023-09-22

**Authors:** Beulah E. R. Samuel, Teresia W. Maina, Jodi L. McGill

**Affiliations:** * Department of Veterinary Microbiology and Preventive Medicine, Iowa State University, Ames, IA; † Cargill Animal Nutrition, Elk River, MN

## Abstract

The bacillus Calmette–Guérin (BCG) vaccine, administered to prevent tuberculosis, is a well-studied inducer of trained immunity in human and mouse monocytes. We have previously demonstrated that aerosol BCG administration induces innate training in calves. The current study aimed to determine whether s.c. BCG administration could induce innate training, identify the cell type involved, and determine whether innate training promoted resistance to bovine respiratory syncytial virus (BRSV) infection, a major cause of bovine respiratory disease in preweaned calves. A total of 24 calves were enrolled at 1–3 d of age and blocked by age into two treatment groups (BCG, *n* = 12; control, *n* = 12). BCG was given s.c. to preweaned calves. The control calves received PBS. We observed a trained phenotype, demonstrated by enhanced cytokine production in response to in vitro stimulation with LPS (TLR-4 agonist) in PBMCs and CD14^+^ monocytes from the BCG group 2 wk (IL-1β, *p* = 0.002) and 4 wk (IL-1β, *p* = 0.005; IL-6, *p* = 0.013) after BCG administration, respectively. Calves were experimentally infected via aerosol inoculation with BRSV strain 375 at 5 wk after BCG administration and necropsied on day 8 postinfection. There were no differences in disease manifestation between the treatment groups. Restimulation of bronchoalveolar lavage fluid cells isolated on day 8 after BRSV infection revealed enhanced IL-1β (*p* = 0.014) and IL-6 (*p* = 0.010) production by the BCG group compared with controls. In conclusion, results from our study show that s.c. administration of the BCG vaccine can induce trained immunity in bovine monocytes and influence cytokine production in the lung environment after BRSV infection.

## Introduction

Cattle are at increased risk of acquiring diseases in early life before their adaptive immune system has fully developed. The United States Department of Agriculture’s National Animal Health Monitoring System reported a 33.8% incidence of morbidity in dairy heifers from birth to weaning (1), with the most common causes of illness being neonatal calf diarrhea and bovine respiratory disease. For decades the livestock industry has relied on broad-spectrum antibiotics to protect young animals from infection (2). This practice has raised public health concerns about antimicrobial resistance in human and animal pathogens and underlines the need for nonantibiotic solutions to control diseases. Immunomodulation of the innate immune system may be an alternative approach to reduce disease severity and provide protection (3).

Several reports have described the capacity of innate immune cells to adapt to previous encounters and produce a heightened immune response, also termed trained immunity (4). Bistoni et al. (5) showed that infection with an attenuated *Candida albicans* strain conferred protection against a challenge with a nonspecific staphylococcal infection. This work was carried out in athymic and RAG-1–deficient mice, supporting that trained immunity can be elicited irrespective of adaptive immunodeficiency. Since then, others have demonstrated that various stimuli can induce trained immunity, such as β-glucans (6), bacterial LPS (7), oxidized low-density lipoprotein (8), and live vaccines such as the bacillus Calmette–Guérin (BCG) vaccine (9).

Although the BCG vaccine is routinely used to prevent severe forms of tuberculosis in humans, epidemiological studies have shown that BCG vaccination can also confer nonspecific protection against unrelated infections (10), which has been attributed to the induction of trained immunity. This has led many to investigate BCG vaccination and the induction of innate training as a strategy to combat other infections. For example, in a study conducted on a group of infants in Guinea-Bissau, Stensballe et al. (11) showed that BCG vaccination promoted a “non-targeted protective effect” against acute lower respiratory tract infection and respiratory syncytial virus (RSV). Arts et al. (12) demonstrated that BCG administration induced nonspecific protection against an experimental yellow fever virus infection in humans, which was correlated with the induction of trained immunity. Kaufmann et al. (13) showed that BCG administration in mice reduced the morbidity and mortality associated with influenza A virus infection due to the induction of more efficient cytokine responses by trained monocytes.

Although there is sufficient evidence of innate training and its benefits in mice and humans, very little work has been done on innate training in agricultural animals. We have previously demonstrated that aerosol BCG administration induces a trained phenotype in bovine PBMCs (14); however, we did not identify which innate cell type was trained. The objective of the current study was to investigate the innate training effects in bovine monocytes after s.c. BCG administration in preweaned calves. Bovine RSV (BRSV) infection is one of the major pathogens that contribute to bovine respiratory disease, particularly in neonatal calves (15). Given the well-described safety profile of BCG and the recent findings demonstrating that BCG-induced training can provide resistance to respiratory diseases in other species, the study’s second objective was to determine whether BCG-induced innate training would protect preweaned calves against BRSV infection.

## Materials and Methods

### Animal care

Neonatal (1–3 d old) colostrum-replete Holstein calves were enrolled in the study. The calves were housed in biosafety level 2 climate-controlled rooms at the Livestock Infectious Disease Isolation Facility at Iowa State University (Ames, IA). On arrival, all calves received a probiotic paste (Achieve) to improve their gut health. The animals had ad libitum access to water, hay, and starter grain and received milk replacer twice daily. Calves were supervised throughout the study by a trained veterinarian. All animal procedures followed Iowa State University Institutional Animal Care and Use Committee guidelines (protocol no. 19-278).

### Study design

A total of 24 male calves were randomly divided into two groups of 12, that is, a treated with BCG group and an untreated control group. The calves were acclimatized for 5 d before administering the BCG vaccine. The treatment group received 1 × 10^7^ CFU of *Mycobacterium bovis* (BCG Danish strain) suspended in 1 ml of PBS s.c. The control group received 1 ml of sterile PBS. The calves were infected with BRSV through the aerosol route 5 wk after receiving BCG. Peripheral blood was collected from the jugular vein after BCG at weeks 2, 4, and 4 d after BRSV challenge. A blinded veterinarian monitored the calves for clinical signs such as body temperature, nasal discharge, eye discharge, expiration effort, and lung sound for 8 d after viral infection. Nasal swabs were collected postchallenge on days 4, 6, and 8 to monitor the nasal viral shedding. At the end of the study, the calves were euthanized by barbiturate overdose, and necropsies were performed.

### BCG administration

*M. bovis* (BCG Danish strain) was cultured as previously described (14). Glycerol stocks of BCG at 5 × 10^7^ CFU/ml concentration were prepared and stored at −70°C. On the day of BCG administration, the glycerol stocks were thawed and reconstituted with PBS to 10^7^ CFU/ml concentration. Each calf received 10^7^ CFU of BCG in 1 ml of PBS s.c. in its right neck. The injection site was monitored for any visible local adverse reaction up to 48 h after BCG administration.

### PBMC and monocyte isolation

Peripheral blood was collected from the calves by jugular venipuncture in a syringe containing 2× acid citrate dextrose solution. Density gradient centrifugation was performed by the overlay of blood diluted 1:1 in PBS on Histopaque-1077 (Sigma-Aldrich, St. Louis, MO), and the PBMCs were isolated from the buffy coat. Hypotonic lysis was carried out to remove contaminating RBCs. Cells were washed twice in PBS, resuspended in complete RPMI 1640 (cRPMI) medium prepared with RPMI 1640 (Life Technologies, Carlsbad, CA) supplemented with 10% (v/v) FBS, 2 mM l-glutamine, 1% antibiotic-antimycotic solution, 1% nonessential amino acids, 2% essential amino acids, 1% sodium pyruvate, 50 μM 2-ME (all from Sigma-Aldrich, St. Louis, MO). The PBMCs were enumerated in the cell counter (Countess II FL automated cell counter, Thermo Fisher Scientific) using a fluorescence-based viability assay.

Monocytes were purified from PBMCs by MACS using CD14 MicroBeads (no. 130-097-052, Miltenyi Biotec). PBMCs were resuspended in 80 μl of MACS buffer (0.5% BSA, 2 mM EDTA in PBS) per 10^7^ cells and 20 μl of CD14 MicroBeads per 10^7^ cells. The cells were incubated at 4°C for 15 min and positively selected by passing through a magnetic column. Cells were washed in PBS, resuspended in cRPMI, and enumerated in the cell counter (Countess II FL automated cell counter, Thermo Fisher Scientific) using a fluorescence-based viability assay.

### BRSV challenge and clinical scoring

BRSV strain 375 was reisolated from an infected lung sample, grown on bovine turbinate cells, passaged fewer than three times, and used for the challenge. As previously described, the calves were infected with ∼10^4^ median tissue culture-infective dose (TCID_50_)/ml BRSV strain 375 via aerosol challenge (16). A blinded veterinarian scored the calves daily for clinical illness following a modified scoring chart from the University of Wisconsin–Madison calf health scoring chart from the day of infection (0 d postinfection) until day 8 postinfection (17). The scoring categories included body temperature, nasal discharge, eye discharge, expiration effort, and lung sounds. One of the calves from the BCG treatment group was euthanized on day 7 postinfection after reaching a humane endpoint. This did not impact data collection except for the samples collected on day 8 postinfection. Therefore, this calf was included in the analyses until the day of the necropsy.

### Necropsy and sample collection

On day 8 after the BRSV infection, the calves were humanely euthanized by barbiturate overdose, and a necropsy was performed. Lungs were collected, the dorsal and ventral sides of the lungs were photographed, and a pathological examination was performed as previously described (16, 18). Eleven lung areas were evaluated for the percentage of lungs affected, and gross pathological scores were assigned using a previously described scoring system (19).

Bronchoalveolar lavage fluid (BALF) was collected by passing 500 ml of ice-cold saline containing 1% antibiotic-antimycotic solution through the trachea and massaging into the lungs. The washout was collected in sterile containers and placed on ice until processing. The BALF was filtered through sterile gauze and centrifuged at 680 × *g* for 10 min to pellet the cells. The cells were washed twice in ice-cold PBS, resuspended in cRPMI, and enumerated by a trypan blue dye exclusion assay on a hemocytometer.

Representative lung tissues from affected (lesioned) and unaffected (nonlesioned) areas were collected in the RNAlater stabilization solution (no. AM7021, Invitrogen) and stored at −70°C for quantifying the lung viral burden through quantitative PCR (qPCR).

### Nasal viral shedding and lung viral burden

Nasal swabs were collected on days 4, 6, and 8 postinfection by swabbing both nostrils with a sterile cotton applicator, and the swabs were stored in serum-free MEM at −70°C until processing. Viral RNA was isolated using the MagMAX viral RNA isolation kit (no. AM1939, Applied Biosystems), and qPCR was performed with a TaqMan RNA-to-CT one-step kit (Applied Biosystems). The viral *NS2* gene copy number was calculated using a known standard curve.

The lung viral burden was determined from the representative lesion and nonlesion lung areas stored in the RNAlater stabilization solution. Viral RNA isolation, cDNA preparation, and qPCR were carried out as previously described (17). Briefly, the viral *NS2* gene was amplified, the gene copy number was calculated using a known standard curve, and the values were normalized to the bovine RPS9 housekeeping gene to correct differences in the input material.

### In vitro stimulation of PBMCs, monocytes, and BALF cells

The cells were plated at 4 × 10^5^ cells per well in a tissue culture–treated 96-well plate. The cells were stimulated with 1 μg/ml *Escherichia coli* B5 LPS (no. tlrl-b5lps, InvivoGen) in cRPMI, 10 μg/ml Pam3CSK4 (no. tlrl-pms, InvivoGen) in cRPMI, or cRPMI alone at 37°C for 48 h. The supernatants were collected by pelleting cells at 470 × *g* for 5 min and stored at −70°C until performing ELISA.

### ELISA

IL-1β and IL-6 ELISAs were performed on the supernatants collected from PBMCs and monocytes following in vitro stimulation using commercial kits (bovine IL-1β ELISA no. ESS0027 and bovine IL-6 ELISA no. ESS0029, Invitrogen). The assays were performed following the manufacturer’s instructions.

### Statistical analysis

GraphPad Prism 9.4.0 was used to generate graphs and perform statistical data comparisons. Statistical significance was determined using two-way ANOVA followed by a Sidak multiple comparison test for the ELISA data. The two-tailed unpaired *t* test was used for gross lung pathology to determine statistical significance. Nasal viral shedding was analyzed using mixed-effects analysis with a Sidak multiple comparison test. The Mann–Whitney *U* test was used to analyze lung viral burden in lesioned and nonlesioned tissue.

## Results

### Enhanced proinflammatory cytokine production in bovine PBMCs and monocytes induced by s.c. BCG administration

**FIGURE 1.**
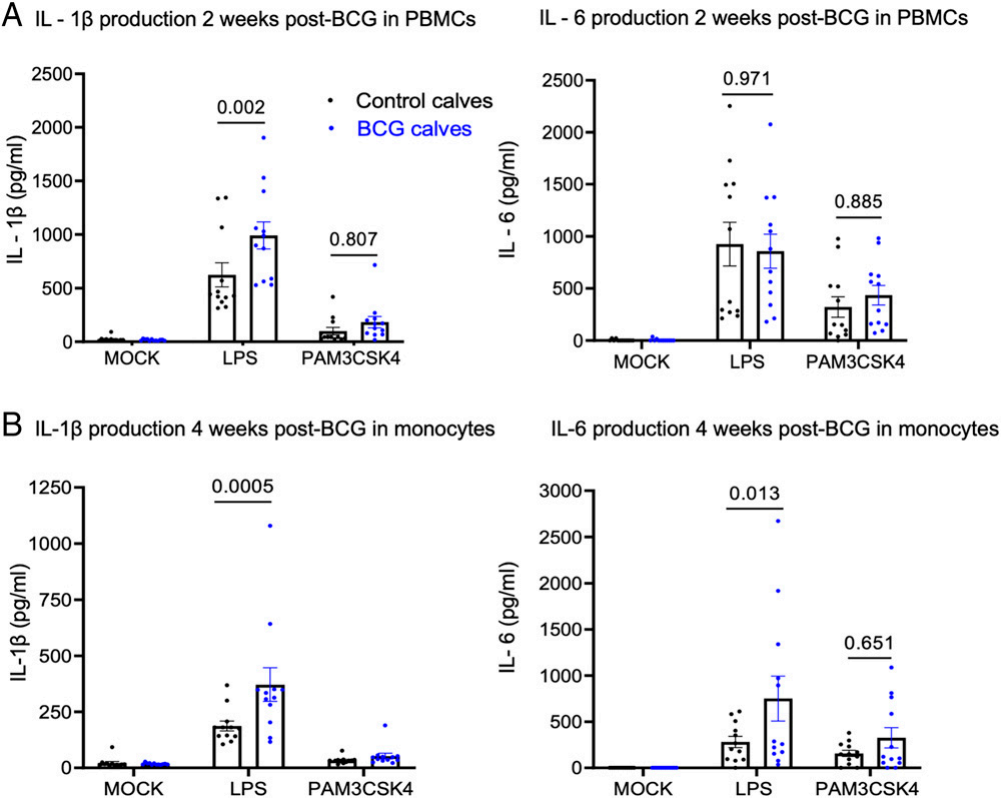
s.c. BCG administration enhances systemic innate cytokine responses in preweaned calves. The BCG group received 1 × 10^7^ CFU of BCG Danish via s.c. injection (*n* = 12). The control group received saline (*n* = 12). (**A**) Peripheral blood was collected 2 wk after BCG administration from calves in both groups and PBMCs were isolated. PBMCs were stimulated in vitro with mock, LPS, or Pam3CSK4 for 48 h. Innate cytokine production was assessed by ELISA on the cell supernatants. (**B**) Peripheral blood was collected 4 wk after BCG administration from calves in both groups and monocytes were separated by MACS sorting using CD14 MicroBeads. Monocytes were stimulated in vitro with mock, LPS, or Pam3CSK4 for 48 h. Innate cytokine production was assessed by ELISA on the cell supernatants. Data are represented as mean ± SEM. The *p* values were determined by two-way ANOVA with a Sidak multiple comparison test.

We have previously reported that aerosol administration of the BCG vaccine induces a trained immune phenotype in calves (14). The current study evaluates innate training responses in preweaned calves following s.c. BCG administration. A total of 24 preweaned Holstein calves were randomly divided into two treatment groups. The BCG group received 1 × 10^7^ CFU of the BCG Danish strain, whereas the control group received PBS only. Peripheral blood was collected at 2 wk after BCG administration, and PBMCs were stimulated in vitro with LPS, Pam3CSK4, or cRPMI (as mock) for 48 h. IL-1β and IL-6 cytokine ELISAs were performed on the supernatants. Consistent with our previous results using aerosol treatment, PBMCs from the calves that received s.c. BCG showed enhanced IL-1β production 2 wk after BCG treatment (*p* = 0.002) on stimulation with LPS (Fig. 1A). However, we observed no differences in IL-6 production between PBMCs from BCG and control calves.

Our previous study demonstrated that BCG priming induces innate training in bovine monocytes in vitro. In the current study, we evaluated trained immune responses exhibited by bovine monocytes exposed to BCG in vivo. We isolated CD14^+^ monocytes at 4 wk after BCG administration and stimulated them with TLR agonists for 48 h, followed by ELISA analysis for IL-1β and IL-6 secretion. Monocyte supernatants from BCG-treated calves showed enhanced IL-1β (*p* = 0.005) and IL-6 (*p* = 0.013) cytokine production compared with supernatants from control calves on stimulation with LPS (Fig. 1B).

### s.c. BCG administration and induction of innate training did not alter the outcome of experimental BRSV infection

**FIGURE 2.**
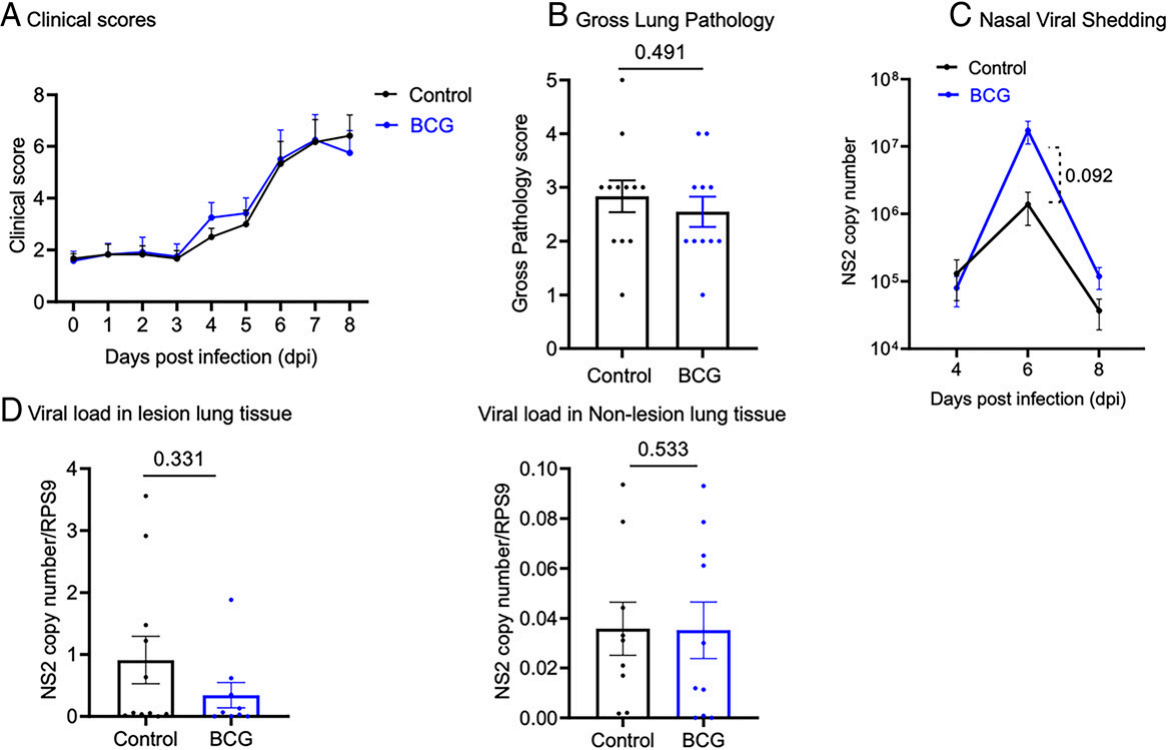
s.c. BCG administration and induction of innate training did not alter the outcome of experimental BRSV infection. Five weeks after receiving s.c. injection, calves were infected with ∼10^4^ TCID_50_/ml BRSV strain 375 via aerosol challenge. (**A**) Clinical scores. Calves in the two groups were monitored daily by a blinded observer and assigned a clinical score using the criteria outlined in *Materials and Methods*. Data are represented as mean ± SEM. (**B**) Gross lung pathology. All animals were humanely euthanized on day 8 postinfection. The extent of gross pneumonic consolidation was evaluated based on the percent of lung affected (0, free of lesions; 1, 1–5% affected; 2, 5–15% affected; 3, 15–30% affected; 4, 30–50% affected; 5, >50% affected). Data are represented as mean ± SEM. The *p* value was determined by a two-tailed unpaired *t* test. (**C**) Nasal viral shedding. Nasal swabs were collected on days 4, 6, and 8 postinfection. Viral isolation and viral quantification by qPCR for the BRSV NS2 gene was performed, and viral NS2 copy numbers were calculated using a standard curve. The *p* value was determined by mixed-effects analysis with a Sidak multiple comparison test. (**D**) Viral load in lung. Lesioned and nonlesioned lung tissue was collected at necropsy. Viral RNA isolation and quantification by qPCR was performed, and lung tissue were normalized to the housekeeping gene RPS9 to correct for differences in input material. Viral NS2 copy numbers were calculated using a standard curve. Data are represented as mean ± SEM. The *p* values were determined by a Mann–Whitney *U* test.

We next evaluated whether innate training induced by BCG administration altered the outcome of an experimental BRSV infection. Calves were challenged via aerosol inoculation of ∼10^4^ TCID_50_ BRSV strain 375 at 5 wk after BCG administration. The calves were monitored daily for clinical signs such as rectal temperature, nasal discharge, eye discharge, expiration effort, and lung sound for 8 d after BRSV infection. Both groups of calves developed clinical signs starting on days 3–4 postinfection, which peaked at 6–7 d postinfection. We observed no differences in clinical signs between the control and treatment groups (Fig. 2A). One of the calves from the BCG treatment group was euthanized on day 7 postinfection after reaching a humane endpoint. At necropsy on day 8 postinfection, the lungs were scored for gross lesions as previously described (19). Gross lung pathology scores did not differ between the treatment groups (*p* = 0.491) (Fig. 2B).

Nasal viral shedding was monitored by collecting nasal swabs at 4, 6, and 8 d after BRSV challenge and assessing viral load by qPCR for the BRSV *NS2* gene. Virus shedding peaked on day 6 for both groups and declined on day 8. No differences were observed between the treatment groups on days 4 or 8 (*p* = 0.924 and *p* = 0.285, respectively); however, control calves tended to shed less virus on day 6 postinfection (*p* = 0.092) (Fig. 2C). Lung viral burden was evaluated in the lesion and nonlesion lung tissue on day 8 postinfection. Viral RNA was detected in the lesion lungs of all calves. In the nonlesion lung samples, viral RNA was detected in all control calves, but only 9 out of 11 calves had detectable levels in the BCG-treated group. However, we observed no differences (lesion lung, *p* = 0.331; nonlesion lung, *p* = 0.533) in lung viral load between the control and treatment groups (Fig. 2D).

### Increased proinflammatory cytokine production in airways after BRSV challenge in BCG-treated calves

**FIGURE 3.**
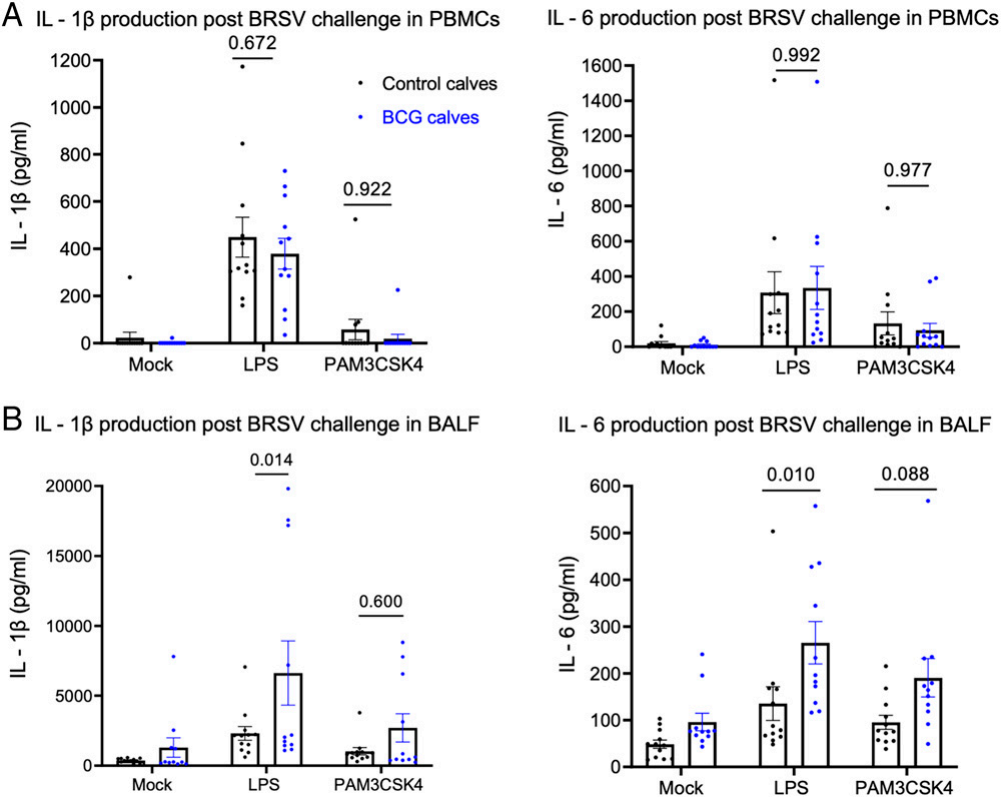
Increased proinflammatory cytokine production by BALF after BRSV challenge in BCG-treated calves. (**A**) Peripheral blood was collected 4 d after BRSV challenge from calves in both groups and PBMCs were isolated. PBMCs were stimulated in vitro with mock, LPS, or Pam3CSK4 for 48 h. Innate cytokine production was assessed by ELISA on the cell supernatants. (**B**) All animals were humanely euthanized on day 8 postinfection. BALF was collected from control (*n* = 12) and BCG (*n* = 11) groups. BALF cells were stimulated in vitro with mock, LPS, or Pam3CSK4 for 48 h. Innate cytokine protein expression was assessed by ELISA on the cell supernatants. In (A) and (B), data are represented as mean ± SEM. The *p* values were determined by two-way ANOVA with a Sidak multiple comparison test.

To determine the impact of BRSV infection on systemic innate immune responses, we evaluated in vitro cytokine production in PBMCs 4 d after BRSV challenge on stimulation with LPS, Pam3CSK4, or cRPMI (as mock) for 48 h. In contrast to the enhanced immune response we observed before infection, we observed no difference in cytokine production by PBMCs collected and stimulated with LPS following BRSV infection (IL-1β, *p* = 0.672; IL-6, *p* = 0.992) (Fig. 3A). Cells from BALF were stimulated in vitro for 48 h with LPS, Pam3CSK4, or cRPMI (as mock). IL-1β and IL-6 ELISAs were performed on the supernatants. Interestingly, we observed enhanced cytokine production by LPS-stimulated BAL cells from BCG-treated calves compared with the control calves (IL-1β, *p* = 0.014; IL-6, *p* = 0.010) (Fig. 3B).

## Discussion

In our previously published work, we demonstrated that aerosol BCG administration to weaned calves induced innate training in PBMCs and that the trained immune phenotype was detectable up to 12 wk after exposure to BCG (14). Importantly, although we demonstrated that training could be induced in PBMCs, the specific cell types impacted by in vivo BCG treatment were not identified. Reports from humans and mice have shown that BCG treatment can train CD14^+^ circulating monocytes (9, 20). However, innate training has subsequently been observed in numerous other cell types, including NK cells (21), neutrophils (22), hematopoietic stem cells (23), and pulmonary epithelial cells (24). In agreement with human studies, our previous work demonstrated that bovine monocytes could be trained in vitro with BCG (14). In the current study, we definitively identify CD14^+^ monocytes as a cell population that is trained in vivo following s.c. BCG administration in calves. Future studies will consider the role of other innate cell populations and their importance in BCG-induced training.

Calves are at particularly high risk from enteric and respiratory infections in the first few weeks. Neonatal calf diarrhea accounts for up to 50% of preweaned dairy heifer deaths in the United States, according to United States Department of Agriculture Dairy 2007 report, while respiratory disease is the second leading cause of death in preweaned animals (25). Given our goal of promoting disease resistance in early life, it is crucial to induce an enhanced immune response state as rapidly as possible after BCG administration to reduce the risk of young animals developing a harmful infection. In adult human trials, there is evidence of innate training as early as 2 wk after receiving the BCG vaccine (20). Similarly, the current study detected evidence of innate training in PBMCs from neonatal calves as early as 2 wk after BCG administration. A signature of trained immunity, and what differentiates innate memory from immune priming or short-lived innate immune activation, is the presence of a heightened response after the immune system has returned to a steady state and an enduring capacity for this heightened response (26). Studies using BCG have reported innate training responses enduring up to 4 wk in low-birth-weight infants (27) and as long as 1 y in adult humans (28). In the current study, we observed BCG-induced trained responses that endured for at least 4 wk in neonatal calves, although our prior work in weaned calves observed that innate training in mixed PBMCs endured for at least 12 wk (14). With this potential protection window, it is feasible that BCG-induced training may reduce disease prevalence in calves through the entire preweaning period.

Multiple studies have explored different routes of BCG administration for the induction of trained immunity. Intradermal vaccination is the predominant route in humans and has been shown repeatedly to induce training in circulating human monocytes (20). Vierboom et al. (29) compared mucosal, intradermal, and i.v. routes and showed that the mucosal route was the best for potent induction of trained immunity in nonhuman primates. Similarly, our previous work demonstrated that the aerosol administration of BCG induces innate training in young calves, although we did not compare it to other routes of administration (14). In calves, aerosol inoculation can be time-consuming and labor-intensive, and administration of i.v. injections requires additional expertise. Jeyanathan et al. (30) showed in a mouse model that s.c. BCG administration trained circulating monocytes and induced trained immunity in the lung via alteration of intestinal microbiota. Therefore, we investigated the s.c. route of BCG administration in our current study. The s.c. layer of skin consists of adipose tissue with fewer blood vessels, which helps in the sustained release of administered substances into the bloodstream and facilitates exposure to circulating monocytes and, subsequently, the hematopoietic stem cells in the bone marrow (31). As we have shown in the current study, s.c. BCG administration also induced a trained immune phenotype in bovine PBMCs and CD14^+^ monocytes. Although it is difficult to determine whether this route is more potent because we did not directly compare routes, the practicality of s.c. over aerosol administration is an essential factor for future applications.

We observed considerable variation in the cytokine responses of individual calves (Figs. 1, 3) and the capacity for trained responses despite efforts to keep host factors consistent, such as single-source procurement of cattle, enrollment of calves of the same age, and control of environmental and nutritional factors. This differential response is similar to the variable protection observed in humans receiving the BCG vaccine (32). The immune responses to BCG vary due to host and environmental factors (33). Brandt et al. (33) showed that prior exposure to environmental mycobacteria blocked the replication of BCG and subsequent induction of protective immunity to tuberculosis in mice. Koeken et al. (32) demonstrated that a differential abundance of circulating metabolites in baseline plasma affected the trained immune responses after BCG administration. This study identified high and low responders based on fold change in ex vivo IL-1β production in response to *Staphylococcus aureus* 3 mo after BCG vaccination. High responders had close to a 2-fold increase, whereas low responders showed a <1-fold increase in cytokine production compared with preimmunization responses. Those termed high responders had an enrichment of metabolites associated with the TCA cycle, glutamine metabolism, and taurine metabolism compared with low responders. Although these environmental and host factors likely contributed to the varied capacity for trained immune responses in our calves, we cannot also rule out the role of epigenetic effects. Verma et al. (34) observed distinct DNA methylation patterns in responders to the BCG vaccine correlating to the enrichment of gene promoters belonging to immune pathways, not seen in nonresponders. A recent study demonstrated the transmittance of epigenetic modifications and associated phenotypes across multiple generations in mammals (35). This suggests a potential role of transgenerational epigenetic effects in immune responses to BCG administration.

There is evidence of trained immunity conferring protection against yellow fever virus infection in humans (12) and BCG protecting human ACE2-transgenic mice from a lethal challenge with SARS-CoV-2 (36). In a study with mice and hamsters, BCG administration protected influenza A virus–infected but not SARS-CoV-2–infected animals (13). They observed that tissue tropism of SARS-CoV-2 to pulmonary endothelial cells leads to lung hemorrhage and dissemination of the virus to bone marrow, suggesting a possibility of SARS-CoV-2 erasing the epigenetic imprinting of BCG in the hematopoietic stem and progenitor cells in the bone marrow. BCG vaccination has been reported to have some beneficial effects against RSV infections in humans. In a population of 386 infants in Guinea-Bissau, girls receiving the BCG vaccine were significantly less likely to develop RSV infection than were unvaccinated controls (11). There was also a tendency for BCG-vaccinated boys to be more resistant to RSV. Thus, there is potential for BCG to promote resistance to RSV. Despite the effective induction of training in calves in the current study, we did not observe increased disease resistance to the BRSV challenge in our BCG-treated calves. In fact, we observed an increase in the amount of virus shed by BCG-treated calves compared with control calves on day 6 postinfection. This is in direct contrast to some of our previous work. In a recent trial investigating a BCG-based vaccine for RSV, Díaz et al. (37) observed that calves receiving BCG tended to develop less lung pathology and reduced clinical scores. Importantly, however, note the difference between the two studies. In the previous study the calves were given two doses of BCG vaccine prior to BRSV challenge, whereas in the current study the calves received just one dose of BCG. Díaz et al. challenged the calves 2 wk after the second dose of BCG, whereas in the current study there was 5 wk between the time of immunization and the challenge. Although we have results from only two studies so far to observe how duration between immunization and time of challenge plays a role in protection against disease, it is a key point to consider in future studies. Another possible explanation for the disparity in our results, in addition to the difference in BCG dose and duration between time of immunization and viral challenge, may be related to the individual variability in responses to BCG, driven by factors such as the abundance of circulating metabolites, epigenetic factors such as DNA methylation, or transfer of epigenetic modification from the parents. Combined with our small sampling size when compared with human trials, it is also possible that we lack the necessary statistical power to consistently detect a benefit from BCG immunization.

In conclusion, we have demonstrated that s.c. BCG administration to preweaned calves induces a trained immune phenotype in circulating CD14^+^ monocytes, which is in consensus with results from human and mouse studies and our own prior in vitro studies from calves. However, in the current trial, we did not observe evidence that innate training promotes increased resistance to BRSV challenge in neonatal calves. Because epigenetic reprogramming of innate immune cells underlies the induction of trained immunity, our future focus will be to explore the epigenetic modifications that regulate innate training in the immune system of young calves. The findings from this study also lead us to consider exploring BCG-induced innate training in the other cell types in cattle and to evaluate the potential nonspecific protective role of BCG in combating other livestock diseases.
